# Next Generation of Computationally Optimized Broadly Reactive HA Vaccines Elicited Cross-Reactive Immune Responses and Provided Protection against H1N1 Virus Infection

**DOI:** 10.3390/vaccines9070793

**Published:** 2021-07-16

**Authors:** Ying Huang, Monique S. França, James D. Allen, Hua Shi, Ted M. Ross

**Affiliations:** 1 Center for Vaccines and Immunology, University of Georgia, Athens, GA 30602, USA; yhuang0@uga.edu (Y.H.); jdallen@uga.edu (J.D.A.); hua.shi1@uga.edu (H.S.); 2 Poultry Diagnostic and Research Center, Department of Population Health, University of Georgia, Athens, GA 30602, USA; mfranca@uga.edu; 3 Department of Infectious Diseases, University of Georgia, Athens, GA 30602, USA

**Keywords:** influenza virus, H1N1 virus, vaccination, protection, immune responses, mice

## Abstract

Vaccination is the best way to prevent influenza virus infections, but the diversity of antigenically distinct isolates is a persistent challenge for vaccine development. In order to conquer the antigenic variability and improve influenza virus vaccine efficacy, our research group has developed computationally optimized broadly reactive antigens (COBRAs) in the form of recombinant hemagglutinins (rHAs) to elicit broader immune responses. However, previous COBRA H1N1 vaccines do not elicit immune responses that neutralize H1N1 virus strains in circulation during the recent years. In order to update our COBRA vaccine, two new candidate COBRA HA vaccines, Y2 and Y4, were generated using a new seasonal-based COBRA methodology derived from H1N1 isolates that circulated during 2013–2019. In this study, the effectiveness of COBRA Y2 and Y4 vaccines were evaluated in mice, and the elicited immune responses were compared to those generated by historical H1 COBRA HA and wild-type H1N1 HA vaccines. Mice vaccinated with the next generation COBRA HA vaccines effectively protected against morbidity and mortality after infection with H1N1 influenza viruses. The antibodies elicited by the COBRA HA vaccines were highly cross-reactive with influenza A (H1N1) pdm09-like viruses isolated from 2009 to 2021, especially with the most recent circulating viruses from 2019 to 2021. Furthermore, viral loads in lungs of mice vaccinated with Y2 and Y4 were dramatically reduced to low or undetectable levels, resulting in minimal lung injury compared to wild-type HA vaccines following H1N1 influenza virus infection.

## 1. Introduction

Influenza is an acute respiratory infection caused by a virus, belonging to the *Orthomyxoviridae family*, which circulates in all parts of the world. Seasonal influenza viruses represent a year-round disease burden, causing illnesses that range in severity, leading to hospitalization and numerous deaths worldwide [1,2]. Globally, an average of 389,000 deaths were estimated to be associated with influenza virus infections each year during the period of 2002–2011 [3]. In the United States, 9–45 million people have been infected with seasonal influenza viruses annually since 2010, resulting in 140,000 to 810,000 hospitalizations and 12,000 to 61,000 deaths (https://www.cdc.gov/flu/anout/burden/, accessed on 12 April 2021). Additionally, the economic burden of influenza virus infection is ~$5.8 billion each year [3,4]. Overall, vaccination is still the most effective intervention to prevent and control influenza virus infection.

Annual seasonal influenza virus vaccines are typically composed of two influenza A virus (IAV) strains representing the A(H1N1) and A(H3N2) subtypes, and either one or two influenza B virus (IBV) strains representing either the Victoria or Yamagata lineages [5,6,7]. Seasonal vaccines predominantly target the continually evolving globular head of the hemagglutinin (HA) protein, and their efficacy has varied between 19% and 60% from 2009 to 2020 [8] depending on the similarity between the antigens in the vaccine and those present in circulating influenza strains [9,10]. Influenza virus vaccine strains are predicted based on annual viral surveillance and released for manufacturing 6 months before the influenza virus seasons begins; therefore, the mismatch of vaccine strains and circulating viruses is highly possible. Influenza virus vaccine efficacy declines whenever an antigenic shift or drift occurs between the vaccine strain recommendation and the influenza viruses in circulation during a given season. Even though the vaccine strains are similar with circulating influenza viruses, the effectiveness of seasonal vaccines are usually between 40% and 60% [11].

In order to overcome antigenic drift and shift, and improve influenza vaccine efficacy, our group has developed a methodology, termed computationally optimized broadly reactive antigens (COBRA) to design HA immunogens for different subtypes of IAVs, including H1, H2, H3, and H5 [12,13,14,15,16,17,18,19]. This COBRA methodology employs multiple rounds of layered consensus building to generate influenza virus vaccine HA antigens that are capable of eliciting broadly reactive HA-specific antibodies that protect against both seasonal and pandemic influenza virus strains [15,16,17,20]. These vaccine immunogens have also inhibited viral infection and virus-induced pathogenesis in mice, ferrets, and non-human primates [21,22,23,24].

Previously, P1 and X6, two historical COBRA HA vaccines, were designed using the traditional COBRA methodology. These HA antigens elicit broadly reactive antibodies with hemagglutination inhibition (HAI) activity against both historical seasonal and pandemic-like H1N1 influenza viruses isolated from humans and swine [12,20]. However, COBRA P1 and X6 HA vaccines typically elicit HAI reactive antibodies against H1N1 viruses from 1933 to 2012, but not against H1N1 viruses that circulated after 2012. Therefore, it was pivotal to generate new COBRA HA vaccines so that they elicit broadly reactive antibodies against currently circulating pandemic-like strains, and also neutralize isolates across multiple future flu seasons.

Previous COBRA design methodologies focus on generating antigens that are broadly reactive against historical and contemporary influenza virus vaccine strains [7,12,17,24]. An emphasis was placed on designing the vaccines using historical influenza isolates, viruses from specific antigenic eras, or past outbreaks. In this study, a new seasonal-based methodology that focused on current and recent circulating viruses was used to update these broadly reactive HA vaccines to better represent the antigen diversity among currently circulating viruses [7]. Using this new methodology, two promising next generation H1N1 COBRA HA vaccine candidates, Y2 and Y4, were generated to elicit antibody responses against a panel of H1N1 viruses isolated from 1983 to 2021. Each COBRA HA antigen was either expressed as soluble trimerized HA proteins or on virus-like particles (VLPs) as immunogens for testing their efficacy in various strains of mice. These two candidates were used in a prime-boost-boost regimen to evaluate their protective efficacy, antibody eliciting potency, and the ability to ameliorate lung injury and inflammation compared to wild-type or historic COBRA H1N1 HA vaccines.

## 2. Materials and Methods

### 2.1. Influenza Viruses

H1N1 Influenza viruses used in this study include: A/Chile/1/1983, A/Singapore/6/1986, A/Beijing/262/1995, A/New Caledonia/20/1999, A/California/07/2009, A/Brisbane/02/2018, and A/Guangdong-Maonan/SWL 1536/2019. All were obtained from Virapur (NY) or BEI Resources. Each virus was amplified in embryonated chicken eggs.

### 2.2. Vaccine Design, Preparation, and HA Content Quantification

Full length wild-type influenza A(H1N1) HA protein amino acid sequences from 6232 human H1N1 viruses collected from May 1, 2013, to April 30, 2019, were downloaded from the Global Initiative on Sharing Avian Influenza Data (GISAID) EpiFlu online database. Sequences were organized by their date of collection and used to produce consensus sequences based on the next-generation COBRA design methodology as previously described [7]. The secondary consensus sequences were then input into 2 different COBRA consensus building scenarios as previously described [7] to generate the final H1 consensus sequences. This process generated 4 HA consensus sequences, 2 of which (Y2 and Y4) were unique. Y2 was derived from sequences isolated between 1 May 2014, and 30 September 2016, and Y4 was derived from sequences collected between 1 October 2016, and 30 April 2019. All full-length HA sequences included in this study and the multiple sequence alignment were shown in Table 1.

Virus-like particles (VLPs) were generated from human embryonic kidney (HEK) 293T cells following DNA plasmid transient transfection as previously described [20,25]. Each VLP contained either a COBRA or wild-type HA antigen plus a neuraminidase from A/mallard/Alberta/24/2001, (H7N3), and the HIV p55 Gag sequences. VLPs were then purified by ultracentrifugation, and then quantified on a 10% SDS-PAGE that was transferred to PVDF membrane. The blot was probed using anti-HA antibodies as previously described [20,25].

Soluble HA proteins were obtained by transfecting truncated HA genes that were cloned into the pcDNA3.1+ plasmid into HEK293T suspension cells as previously described [26]. The truncated HA genes were generated by replacing the transmembrane domain with a T4 fold-on domain, an Avitag, and a 6× His-tag for purification [26]. The concentration of the soluble HA proteins was determined by conventional bicinchoninic acid assay (BCA) according to the manufacture’s instruction.

### 2.3. Animal Vaccination and Infection

BALB/c and DBA/2J mice (females, 6 to 8 weeks old) were purchased from Jackson Laboratory (Bar Harbor, ME, USA), housed in microisolator units, and allowed free access to food and water; they were cared for under USDA guidelines for laboratory animals. All procedures were reviewed and approved by the University of Georgia Institutional Animal Care and Use Committee (IACUC) (no. A2018 06-018-Y3-A16). Eighty-eight BALB/c mice were randomly divided into 8 groups, with 11 mice in each group. Mice were vaccinated intramuscularly with either 1 μg of COBRA P1, X6, Y2, Y4, Brisbane/59/2007, California/07/2009, Brisbane/02/2018 VLPs or PBS formulated with AddaVax (oil-in-water emulsion) (InvovoGen, San Diego, CA, USA) at a 1:1 ratio for a final volume of 50 μL. At weeks 4 and 8 following the first vaccination, mice were boosted with the same amount of VLPs or PBS intramuscularly.

Another set of 64 DBA/2J mice were divided into 8 groups (*n* = 8/group) and were vaccinated with 1 μg of the corresponding soluble recombinant HA proteins mentioned above using the same vaccination regimen. Blood was collected at weeks 6 and 10 following the first vaccination and sera were separated and stored at −20 °C for future use. At week 12, all mice were infected with 5 × 10^4^ PFU of wild-type H1N1 A/California/07/2009 (CA/09) or 8.75 × 10^6^ PFU of H1N1 A/Brisbane/02/2018 (Bris/18) via intranasal route in a volume of 50 μL. Mice were monitored and their body weights were recorded daily for 14 days post infection. At days 3 and 6 post infection, three mice from each group were sacrificed and the lungs were collected, the left lung was inflated with 10% neutral formalin for histopathology, and the right lung lobes were snap-frozen on dry ice and then stored at −80 °C for assessing virus titers. Mice were humanely euthanized once they reached humane endpoints by losing 20% of their original body weight or accumulated a clinical disease score of 3 as previously described [20]. All procedures were performed in accordance with Guide for the Care and Use of Laboratory Animals, the Animal Welfare Act, and Biosafety in Microbiological and Biomedical Laboratories.

### 2.4. Enzyme-Linked Immunosorbent Assay (ELISA)

ELISA was used to assess antibody reactivity against different H1N1 HA strains and performed as previously described [27]. In brief, Immulon 4HBX plates (Thermo Fisher Scientific, Waltham, MA, USA) were coated at 4 °C overnight with 50 μL per well with a solution of carbonate buffer (pH 9.4) containing 1 μg/mL of the different rHAs (A/California/07/2009, A/Brisbane/02/2018), or cH6/1 purified rHA and 5 μg/mL of bovine serum albumin (BSA) in a humidified chamber. An amount of 5 μg/mL BSA (50 μL per well) was coated alone as a negative control. Plates were blocked with ELISA blocking buffer in a volume of 200 μL/well for 1 h at 37 °C. Serum samples were serially diluted 3-fold in blocking buffer starting from a dilution of 1:100, and then added into HA protein coated plates. After incubation at 4 °C overnight, the 1:2000 diluted goat anti-mouse IgG (Southern Biotech, Birmingham, AL, USA) secondary antibody was added into each well in a volume of 100 μL, and incubated at 37 °C for 1 h. Finally, 50 μL of ABTS substrate (VWR Corporation) was added into each well and further incubated at 37 °C for 15–20 min. Colorimetric conversion was terminated by adding 50 μL of 1% SDS into each well. The O.D. values (OD 414) were measured by a spectrophotometer (PowerWave XS, BioTek) at 414 nm.

### 2.5. H&E Staining

For pathological analysis, lung sections were respectively subjected to H&E staining. Three mice from each group were anesthetized and perfused with 10% neutral buffered formalin followed by PBS on day 3 post infection. Left lungs were removed and fixed in 10% formalin for another 7 days before being subjected to paraffin embedding. Then, 5 µm thick transverse sections were mounted in Apex superior adhesive slides (Leica biosystem Inc., Buffalo Grove, IL, USA) that were coated to have a positive charge. Sections were deparaffinized in Xylene and hydrated using different concentrations of ethanol (100%, 95%, 80% and 75%) for 2 min each. Deparaffinized and hydrated lung sections were stained with Hematoxylin (MilliporeSigma, Burlington, MA, USA) for 8 min at RT, differentiated in 1% acid alcohol for 10 s, and then counterstained with Eosin (MilliporeSigma, Burlington, MA, USA) for 30 s. Slides were then dehydrated with 95% and 100% ethanol, cleared by Xylene, and mounted using Permount^®^ mounting media (Thermo Fisher Scientific, Waltham, MA, USA). Lungs were scored for pathology following a previously described method [28].

### 2.6. Plaque Assay

MDCK cells within 20 passages were seeded in each well of a six-well plate at a concentration of 1 × 10^6^ cells/well one day prior to performing the plaque assay. Frozen Lung tissues were thawed on ice and homogenized in 1 mL of DMEM. Homogenate was centrifuged at 2000 rpm for 5 min to remove tissue debris, and the supernatant was collected and subjected a serial 10-fold dilution in DMEM supplemented with 1% penicillin-streptomycin. MDCK cells with a 90% confluency in each well were infected with 100 μL of each dilution of homogenate supernatant. The plates were then shaken every 15 min for 1 h. After 1 h incubation, the supernatant was removed and cells were washed twice with fresh DMEM. Finally, 2 mL of 2× MEM and 0.8% agarose overlay (Cambrex, East Rutherford, NJ, USA) was added into each well, and the plates were incubated at 37 °C with 5% CO_2_ for another 72 h. After that, overlay was removed from each well, and the cells were fixed with 10% buffered formalin for 20 min and stained with 1% crystal violet (Fisher Science Education, Waltham, MA, USA) for 15 min at room temperature (RT). Plates were then rinsed thoroughly using tap water to remove excess crystal violet. The plaques were enumerated, and the lung viral titers were calculated and presented as PFU/mL.

### 2.7. Hemagglutination Inhibition Assay (HAI)

The HAI assay was used to evaluate functional antibodies binding to HA protein that are capable of inhibiting red blood cell agglutination. This protocol was adapted from the WHO laboratory influenza surveillance manual [29]. In this study, HAI assays were performed against a panel of 7 H1N1 influenza viruses, including: A/Chile/1/1983, A/Singapore/6/1986, A/Beijing/262/1995, A/New Caledonia/20/1999, A/California/07/2009, A/Brisbane/02/2018, and A/Guangdong-Maonan/SWL 1536/2019. The HAI assay was performed as previously described [12]. Briefly, sera were treated with receptor-destroying enzyme (RDE) (Denka Seiken, Co., Tokyo, Japan) prior to being tested to remove nonspecific inhibitors by incubating overnight at 37 °C, the RDE was then further inactivated at 56 °C for 45 min. An amount of 25 μL of PBS was added to a 96-well V-bottom plate in row 2–12, 50 μL RDE-treated sera was added into row 1, and then a 2-fold serial dilution was performed across the plate. An equal volume of H1N1 virus with approximately 8 hemagglutination units (HAU)/50 μL was added into each well. The plates were incubated at RT for 30 min, and then a solution of 0.8% turkey erythrocytes in PBS were added in a volume of 50 µL to each well. The plate was mixed by agitation and incubated at RT for another 30 min. The HAI titer was determined as the reciprocal dilution of the last well that contained non-agglutinated RBCs. Positive and negative serum controls were included for each plate. An HAI titer greater than 1:40 was defined as seroprotective, and a 4-fold increase in HAI titer compared to the baseline was considered seroconversion in accordance with the WHO and European Committee for Medicinal Products guidelines to evaluate influenza vaccines [30].

### 2.8. Focus Reduction Assay (FRA)

The FRA used in this study was initially developed by the World Health Organization collaborating center in London, UK [31,32], and modified by U.S. Centers for Disease Control and Prevention (CDC). Sera samples were treated with RDE as described above. Initially, 100 μL of MDCK cells at a concentration of 3 × 10^5^ cells/mL were seeded into each well in a 96-well flat-bottom plate. After 24 h, the cells were allowed to reach 95% to 100% confluency and were then washed with PBS. Next, 50 μL of 2-fold serial diluted sera samples were added to each well staring with a 1:20 dilution in virus growth medium (VGM) supplemented with 1 μg/mL tosylsulfonyl phenylalanyl chloromethyl ketone (TPCK)-treated trypsin (VGM-T) (Sigma, St. Louis, MO, USA). Afterwards, influenza virus was diluted in VGM-T, and 50 μL of virus solution at a concentration of 1.2 × 10^4^ FFU/mL was added to each well; VGM-T alone was also added to cell control wells. Plates were incubated at 37 °C for 2 h, and then 100 μL of overlay was added into each well. The overlay consists of equal volumes of 1.2% Avicel RC/CL (FMC Health and Nutrition, Philadelphia, PA, USA) and 2× MEM supplemented with 1 μg/mL TPCK-treated trypsin, 0.1% BSA, and 1% penicillin-streptomycin [31]. After 18–22 h of incubation at 37 °C, the overlay was removed and the cells were washed twice using PBS. Lastly, cells were fixed with ice-cold 4% formalin at 4 °C for 30 min, followed by washing once with PBS and permeabilizing with 0.5% Triton X-100 at RT for 20 min. Monolayers were washed three times with PBS containing 0.1% Tween 20 (wash buffer) and incubated with a mouse-anti-IAV nucleoprotein monoclonal antibody [33] at 37 °C for 1 h. After washing three time with wash buffer, the cells were incubated with a secondary antibody, goat anti-mouse peroxidase-labeled IgG (SeraCare, Inc., Milford, MA, USA), for 1 h at RT. Cells were then washed three times with wash buffer, and TrueBlue substrate (SeraCare, Inc., Milford, MA, USA) containing 0.03% H_2_O_2_ was added and incubated for 10 to 15 min at RT. The reaction was terminated by washing plates with distilled water five times. Plates were air-dried and foci were counted using a CTL BioSpot Analyzer with ImmunoCapture 6.4.87 software (CTL, Cleveland, OH, USA). The FRA titer was presented as the reciprocal of the highest dilution of sera corresponding to 50% focus reduction compared to the virus control wells minus the cell control wells.

## 3. Statistical Analysis

All data are presented as absolute mean values ± standard errors of the means (SEM). One-way ANOVA was used to analyze the statistical differences among groups using GraphPad Prism 9 software (GraphPad, San Diego, CA, USA). A “*p*” value less than 0.05 was defined as statistically significant (*, *p* < 0.05; **, *p* < 0.01; ***, *p* < 0.001; ****, *p* < 0.0001).

## 4. Results

### 4.1. Next Generation H1N1 COBRA Vaccines Protected Mice from Viral Challenge

**Figure 1:**
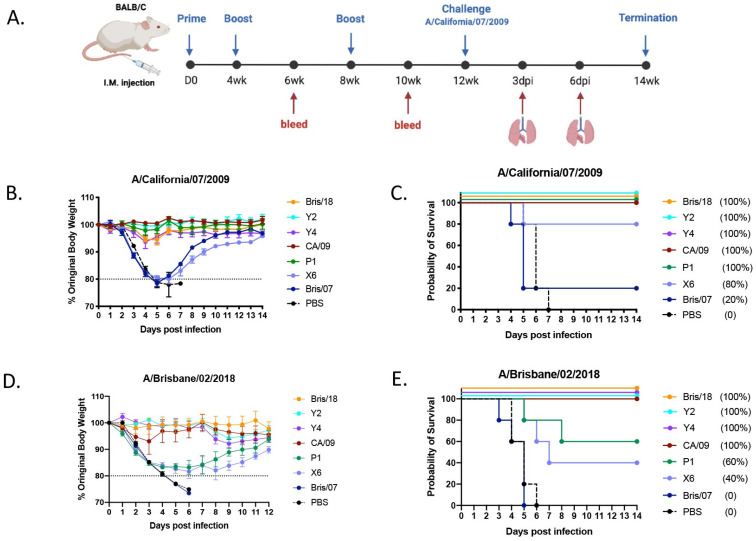
Schematic of animal study. (**A**) Animal study outline: Eighty-eight BALB/c mice (*n* = 11) were intramuscularly vaccinated with COBRA or wild-type HA VLP vaccines formulated with AddaVax adjuvant at weeks 0, 4, and 8. At weeks 6 and 10 post vaccination, blood was collected, and the sera were separated for analysis. At week 12, all mice were inoculated with 5 × 10^4^ PFU of A/California/07/2009 H1N1 virus intranasally, lung tissues (*n* = 3/group) were harvested on days 3 and 6 post infection and evaluated for histopathology and virus titration. (**B**) Body weight loss of mice post infection: The mice were observed for clinical signs for 14 days, and their body weight was recorded daily post infection. The dotted line indicates 80% of their body weights on D0 post infection. (**C**) Survival cure after infection with A/California/07/2009 virus. Another 64 DBA/2J mice were intramuscularly vaccinated with COBRA or wild-type rHA vaccines formulated with AddaVax adjuvant using the same vaccination regimen mentioned above. At week 12, all mice were intranasally infected with 8.75 × 10^6^ PFU of A/Brisbane/02/2018 H1N1 virus. (**D**) Body weight loss curve of DBA/2J mice after infection with A/Brisbane/02/2018 H1N1 virus. (**E**) Survival cure after infection with A/Brisbane/02/2018 virus.

In order to determine the protective efficacy of the next generation H1N1 COBRA HA vaccines, BALB/c mice (*n* = 11/group) were intramuscularly inoculated with purified VLPs expressing either COBRA or wild-type HA antigens (1 μg/HA content) formulated with AddaVax (an oil-in-water-based adjuvant) three times at 4-week intervals. On week 12 following the first vaccination, mice were challenged intranasally with CA/09 H1N1 influenza virus at concentration of 5 × 10^4^ PFU/50 μL (Figure 1A). Mice vaccinated with Y2, Y4, CA/09, and P1 VLPs all survived the CA/09 infection with little to no body weight loss (maximal 5%) (Figure 1B,C). Meanwhile, PBS-vaccinated mice lost their body weight rapidly after day 2 post infection, and reached the humane endpoint by day 7 post infection. 80% of mice vaccinated with the COBRA X6 VLP survived the CA/09 infection (Figure 1C), but they lost around 18% to 20% body weight by day 6 post infection, and then gradually recovered (Figure 1B). Mice vaccinated with Bris/07 VLP vaccine failed to protect against the CA/09 infection as expected (Figure 1B,C).

Next, we further tested the protective efficacy of the next generation H1N1 COBRA vaccines against Bris/18 H1N1 virus challenge. Since BALB/c mice are not very susceptible to Bris/18 infection, we chose another strain of mice (DBA/2J) for challenge. A set of 6–8-week-old DBA/2J mice were vaccinated using the same regimen mentioned above with 1 μg of rHA proteins formulated with AddaVax. On week 12 post vaccination, mice were infected with Bris/18 virus at a concentration of 8 × 10^6^ PFU/50 μL intranasally. Mice vaccinated with Bris/18, Y2 and Y4 rHAs all survived with little or no body weight loss during the 14 day infection (Figure 1D,E). Although the CA/09 rHA-vaccinated mice all survived the Bris/18 challenge, they lost about 10% of their body weight within 3 days post infection, and did not start gaining weight until 5 days post infection. In contrast, mice vaccinated with historic COBRA P1 and X6 rHAs lost 18–20% body weight by day 6 post infection with a 60% and 20% survival rate, respectively. However, all mice vaccinated with Bris/07 rHA or PBS all succumbed to disease and reached their humane endpoint by day 6 post infection.

### 4.2. Next Generation H1N1 COBRA Vaccines Elicited Broader and Higher HAI Titer in Mice

**Figure 2:**
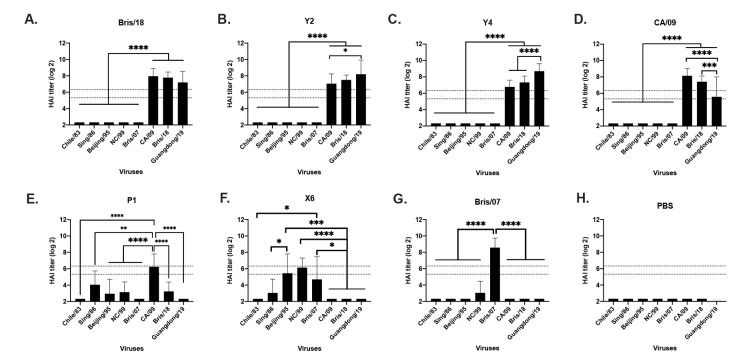
Serum HAI antibody titers post vaccination against a panel of H1N1 viruses. Immunologically naive BALB/c mice were vaccinated three times at 4-week intervals with Y2, Y4, P1, and X6 COBRA H1N1 VLP vaccines or H1N1 wild-type Bris/07, CA/09, or Bris/18 VLP vaccines, and sera were collected on week 10 post first-vaccination for HAI assay against a panel of 7 H1N1 influenza viruses. (**A**) Bris/19; (**B**) Y2; (**C**) Y4; (**D**) CA/09; (**E**) P1; (**F**) X6; (**G**) Bris/07; (**H**) PBS. Y axis indicates the log2 HAI titers for each vaccinated group and presents them as absolute mean values ± SEM. The dotted lines indicate HAI titers ranging from 1:40 (lower line) and 1:80 (upper line). HAI titers were statistically analyzed using nonparametric one-way ANOVA by GraphPad Prism 9 software (GraphPad, San Diego, CA, USA). A *p* value of less than 0.05 was defined as statistically significant (*, *p* < 0.05; **, *p* < 0.01; ***, *p* < 0.001; ****, *p* < 0.0001).

In order to evaluate the antibody breadth elicited by the next generation H1N1 COBRA vaccines, sera were collected from mice at week 10 post initial vaccination. Mice vaccinated with Bris/18 VLPs had antibodies with HAI activity (HAI+) against CA/09, Bris/18 and Guangdong/19 pandemic-like viruses that circulated from 2009 to 2019 (Figure 2A). Mice vaccinated with Y2 and Y4 VLPs had HAI+ antibodies against the CA/09, Bris/18, and Guangdong/19 viruses (Figure 2B,C), Notably, Y2 and Y4 antisera had significantly higher HAI titers against Guangdong/19 than CA/09 and/or Bris/18 viruses (Figure 2B,C). Mice vaccinated with CA/09 VLPs had high titer HAI+ antibodies against both CA/09 and Bris/18, but the HAI titer significantly decreased against the most recently circulated Guangdong/19 virus (Figure 2D). In contrast, mice vaccinated with COBRA P1 VLPs had HAI+ antibodies against both historical seasonal influenza viruses and the pandemic CA/09 virus (Figure 2E), which is consistent with what has been previously reported [12,18], but it was noticed that P1 VLP did not elicit HAI+ antibodies against Bris/18 or Guangdong/19 viruses (Figure 2E). Mice vaccinated with COBRA X6 VLPs had HAI+ antibodies against all seasonal-like H1N1 viruses to a certain degree (Figure 2F), and mice vaccinated with Bris/07 only had antibodies against the homologously matched Bris/07 virus, as expected (Figure 2G). PBS-vaccinated mice did not have any HAI+ antibodies against any viruses (Figure 2H).

### 4.3. Next Generation H1N1 COBRA Vaccines Decreased the Lung Viral Loads after Infection

**Figure 3:**
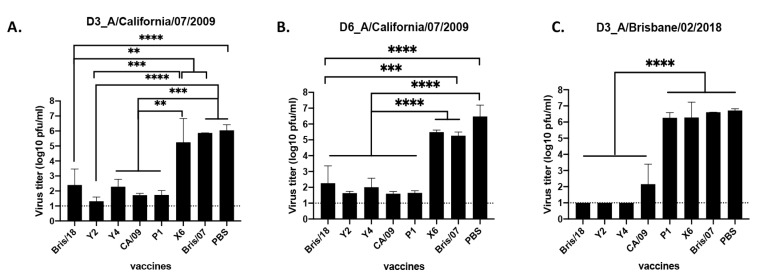
Viral titers in the lung tissues of BALB/c and DBA/2J mice. BALB/c mice were intramuscularly vaccinated with COBRA or wild-type HA VLP vaccines, and then challenged with H1N1 A/California/07/2009 virus at week 12 post vaccination, lung samples (*n* = 3/group) were collected at days 3 and 6 post infection. Lung viral titer on day 3 post infection (**A**) and day 6 post infection (**B**) were determined. Another set of DBA/2J mice with the same vaccines delivered in a rHA format were challenged with A/Brisbane/02/2018. Lung samples (*n* = 3) were harvested on day 3 post infection, and the viral titer was determined (**C**). Viral titers in lung tissue are presented as PFU/mL shown on Y axis. The X axis indicates the different vaccines used in this study. A nonparametric one-way ANOVA was used to analyze statistical differences between groups using GraphPad Prism 9 software (GraphPad, San Diego, CA, USA). A *p* value less than 0.05 was defined as statistically significant (*, *p* < 0.05; **, *p* < 0.01; ***, *p* < 0.001; ****, *p* < 0.0001).

In order to assess the viral titers in lung tissues after infection, three mice from each group were sacrificed on day 3 and day 6 post infection, and the virus titer of CA/09 virus in the lung tissues was evaluated (Figure 3). PBS-vaccinated mice had the highest lung viral titers (1.32 × 10^6^ PFU/mL) on day 3 post infection, and mice vaccinated with Bris/07 and X6 had statistically similar lung viral titers compared to the PBS-vaccinated mice (Figure 3A). However, Y2-vaccinated mice had the lowest lung viral titers (20 pfu/mL), which are statistically similar with that of CA/09 and P1-vaccinated mice (less than 50 pfu/mL), while mice vaccinated with Y4 and Bris/18 HA vaccines had a 4-log decrease in lung viral titers (average of 1.99 × 10^2^ to 2.51 × 10^2^ PFU/mL) compared to mice vaccinated with PBS. Lung viral titers on day 6 post infection were also determined, and the titers were statistically similar with those detected on D3 post infection (Figure 3B).

For the Bris/18 challenge, PBS-vaccinated mice had the highest lung virus titers (∼5.23 × 10^6^ PFU/mL) on day 3 post infection, as expected (Figure 3C). Mice vaccinated with either COBRA Y2, Y4 or Bris/18 had no detectable viral titer in their lungs and mice vaccinated with CA/09 had ∼1.43 × 10^2^ PFU/mL of virus in their lungs, which was significantly lower (4-log decline) than that in PBS-vaccinated mice. However, mice vaccinated with P1, X6, and Bris/07 had statistically similar lung viral titers, and the titers were similar to that in PBS-vaccinated mice (Figure 3C).

### 4.4. Antibodies Elicited by Next Generation of H1N1 COBRA Vaccines Are Mainly against HA Head

**Figure 4:**
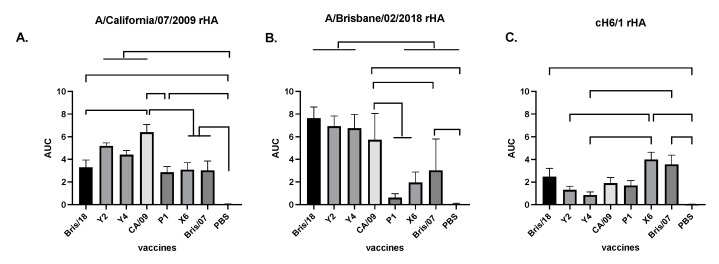
Total IgG antibody responses in mice. Vaccine responses in BALB/c mice were evaluated at week 10 post vaccination with COBRA, wild-type HA VLP vaccines, or PBS formulated with AddaVax. IgG antibody titers were determined against (**A**). A/California/07/2009 HA protein, (**B**) A/Brisbane/02/2018 HA protein, or (**C**) cH6/1 HA protein (Chimeric rHA with globular head from A/California/07/2009 HA and stalk form subtype H6 influenza virus HA). The data is presented as area under curve (AUC) obtained OD141 values from 3-fold serially diluted sera plus SEM. For each independent experiment, mouse sera were assayed in duplicate. One-way ANOVA was used to analyze the statistical differences between groups by GraphPad Prism 9 software (GraphPad, San Diego, CA, USA). A *p* value less than 0.05 was defined as statistically significant (*, *p* < 0.05; **, *p* < 0.01; ***, *p* < 0.001; ****, *p* < 0.0001).

In order to assess the IgG antibodies elicited by COBRA Y2 and Y4 vaccines against the CA/09 and Bris/18 viruses, antisera collected from VLP-vaccinated mice were assessed for binding to CA/09 and Bris/18 soluble rHA antigens (Figure 4). Mice vaccinated with different VLPs had significantly higher IgG antibody titers against CA/09 HA than those in PBS-vaccinated mice (Figure 4A). As expected, antisera from CA/09-vaccinated mice had the highest total IgG titers against the homologously matched CA/09 HA protein and antisera from COBRA Y2- and Y4-vaccinated mice had statistically similar IgG antibody titers compared those in CA/09-vaccinated mice (Figure 4A). Meanwhile, Bris/18-vaccinated mice had lower IgG antibody titer compared to that in CA/09-vaccinated mice (Figure 4A). However, mice vaccinated with P1, X6, and Bris/07 VLPs had significantly lower IgG antibody titer against CA/09 compared to that in CA/09- and Y2-vaccinated mice (Figure 4A), suggesting that the Y2 and Y4 HA antigens on the VLPs efficiently elicited similar IgG titers as those generated by the homologously matched CA/09 HA. However, antisera from mice vaccinated with Y2 and Y4 VLPs had statistically similar total IgG titers against Bris/18 HA compared to that in Bris/18- and CA/09 VLPs-vaccinated mice, while P1-vaccinated mice had the lowest titer against Bris/18 HA (Figure 4B).

Next, we determined if the antibodies induced by the vaccines are head-specific or stalk-specific by using a chimeric HA protein (cH6/1) with a globular head from H1 (CA/09) and the stalk region from the H6 HA protein as previously described [34]. The antibodies elicited by the X6 and Bris/07 vaccines had the highest binding activity against cH6/1 protein (Figure 4C), suggesting that X6- and Bris/07-vaccinated mice had significantly higher stalk-specific antibodies than any other vaccinated mice. In contrast, antibodies elicited by Y2 and Y4 VLP vaccines had the lowest level of stalk-specific antibody compared to any other vaccines (Figure 4C).

### 4.5. Next Generation H1N1 COBRA Vaccines Elicited a High Level of Neutralizing Antibodies against H1N1 Viruses

**Figure 5:**
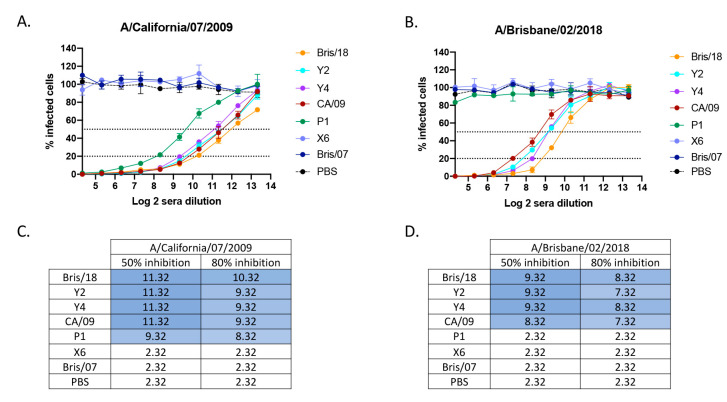
Neutralizing antibody titers in mouse sera post vaccination. Immunologically naive BALB/c mice (*n* = 11/group) were vaccinated three times at 4-week intervals with Y2, Y4, P1, and X6 COBRA H1N1 VLP vaccine or H1N1 wild-type Bris/07, CA/09, or Bris/18 VLP vaccines. At week 10 post vaccination, sera were collected for FRA assay against (**A**) A/California/07/2009 and (**B**) A/Brisbane/02/2018 viruses. For each virus, the virus concentration was standardized to 1.2 × 10^4^ FFU/mL, and the virus alone infected well was standardized as 100% infection. The X axis indicates log 2 sera dilution, and the Y axis represents the percentage of infected cells compared to virus-only infected control wells. The dotted lines represent the 50% inhibition (upper line) and the 80% inhibition (lower line) by the antisera. (**C**,**D**) Heat maps of the log2 serum dilution titers when 50% or 80% of the infection was inhibited against CA/09 (**C**) or Bris/18 virus (**D**). Colors range from white (lowest inhibition) to dark blue (highest inhibition).

To evaluate the neutralizing activity of antibodies elicited by next generation H1N1 COBRA vaccines, a focus reduction assay was used to assess neutralizing titers against challenge virus as well as more recent circulating H1N1 viruses in vitro (Figure 5). Mice vaccinated with PBS, wild-type Bris/07 VLP, and COBRA X6 VLP vaccines did not have detectable neutralizing antibody titers against either CA/09 virus (Figure 5A) or Bris/18 virus (Figure 5B). Mice vaccinated with COBRA Y2 and Y4 VLP vaccines had high neutralizing antibody titers against both CA/09 and Bris/18 viruses. Antisera from COBRA Y2- and Y4 VLP-vaccinated mice had a log2 titer of 11.32 (50% inhibition) against CA/09 virus (Figure 5C) and log 2 of 9.32 (50% inhibition) against the Bris/18 virus (Figure 5D), which was equal to that elicited by their homologously matched HA VLP vaccines. However, mice vaccinated with the COBRA P1 VLP vaccine had high neutralizing titer (50% and 80% inhibition) against the CA/09 virus (Figure 5A,C), but no detectable neutralizing antibody titer against Bris/18 virus (Figure 5B,D). Neutralizing antibodies elicited by the CA/09 vaccine had high cross reactivity with those induced by Bris/18 vaccine. The mice vaccinated with CA/09 had a lower log2 (50% and 80% inhibition) titer against the Bris/18 virus than those in mice vaccinated with the Bris/18 vaccine (Figure 5D), while Y2 and Y4 VLP elicited neutralizing antibody titers against the CA/09 and Bris/18 viruses that were equivalent to those elicited by their homologous vaccines.

### 4.6. Next Generation H1N1 COBRA Vaccines Protect Animals from Infection with Less Injury and Moderate Inflammation

**Figure 6:**
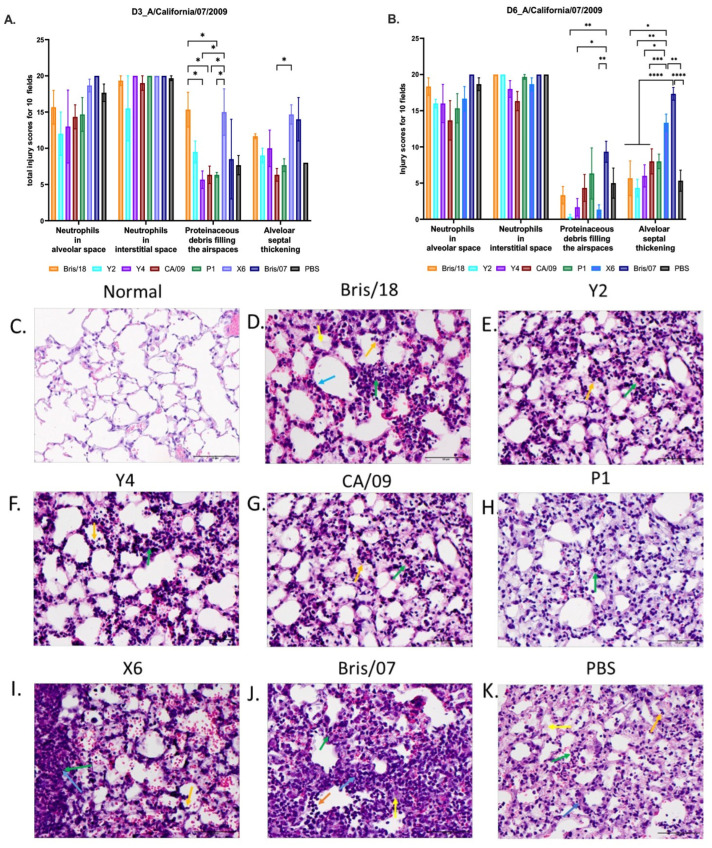
Lung injury in mice after A/California/09/2007 virus infection. Three mice from each group were euthanized on day 3 (**A**) and day 6 post infection (**B**). The left lungs were inflated with 10% buffered formalin and then embedded into paraffin blocks. H&E staining was performed on 3 sectional slides for each mouse lung sample. Neutrophils in alveolar space, neutrophils in interstitial space, proteinaceous debris filling the airspaces, and alveolar septal thickening were assessed. Each parameter was evaluated in 10 random fields under 40× magnification (scale bar indicated 50 µm), and then, the total injury scores of 10 fields were determined. Scoring system: Neutrophils in alveolar space (indicated with orange arrows): 0 = none, 1 = 1–5, 2 = >5; neutrophils in interstitial space (indicated with green arrows): 0 = none, 1 = 1–5, 2 = >5; proteinaceous debris filling the airspaces (indicated with yellow arrows): 0 = none, 1 = 1, 2 = >1; alveolar septal thickening (indicated with blue arrows): 0 = <2×, 1 = 2×–4×, 2 = >4×. (**C**) Normal mouse left lung (no vaccination or infection). (**D**–**K**) Representative images of alveolar septal thickening on day 6 post infection in mice vaccinated with (**D**) Bris/18, (**E**) Y2, (**F**) Y4, (**G**) CA/09, (**H**) P1, (**I**) X6, (**J**) Bris/07, and (**K**) PBS. The data are presented as absolute mean plus SEM. A one-way ANOVA was used to analyze the statistical differences of the lung injury scores using GraphPad Prism 9 software (GraphPad, San Diego, CA, USA). A *p* value less than 0.05 was defined as statistically significant (*, *p* < 0.05; **, *p* < 0.01; ***, *p* < 0.001; ****, *p* < 0.0001).

In order to evaluate the lung injury in the vaccinated mice after infection, mice (*n* = 3/group) were euthanized on days 3 and 6 after CA/09 virus infection. Neutrophils in the alveolar and interstitial space, proteinaceous debris filling the airspaces, alveolar septal thickening, and the extent of lung inflammation were assessed. As shown in Figure 6A,B, mice vaccinated with PBS, Bris/07, and X6 VLP vaccines had the highest lung injury scores when compared to other vaccinated groups.

There was no significant difference in the number of neutrophils detected in alveolar and interstitial spaces among the mice vaccinated with different VLP vaccines on day 3 post infection, but Y2- and Y4 VLP-vaccinated mice had the lowest average number of neutrophils infiltrating the alveolar and interstitial spaces (Figure 6A). However, there was significantly reduced amounts of proteinaceous debris filling the airspaces in Y4, CA/09, and P1 VLP-vaccinated mice compared to that in mice vaccinated with X6 VLP vaccine (Figure 6A). Mice vaccinated with X6 had a significantly higher level of alveolar septal thickening than the mice vaccinated with any other vaccines (Figure 6C–K). Moreover, the same injury trend was also observed in lungs after 6 days post infection, but alveolar septal thickening was significantly decreased in mice vaccinated with Bris/18, Y2, Y4, CA/09, and P1 VLPs compared to mice vaccinated with X6 and Bris/07 VLPs (Figure 6B).

**Figure 7:**
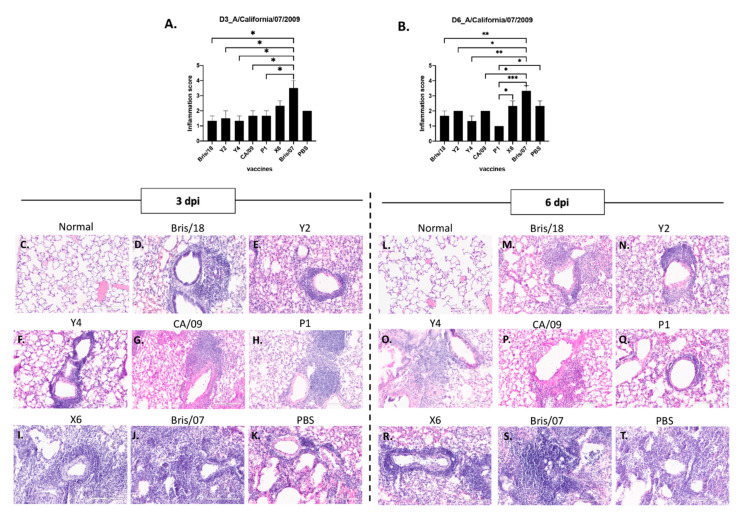
Lung inflammation in mice after A/California/09/2007 virus infection. Three mice from each group were sacrificed at day 3 and 6 post infection; left lungs were inflated with 10% buffered formalin, and then embedded into paraffin blocks. H&E staining was performed on 3 sectional slides for each mouse lung sample. (**A**). The inflammation score of day 3 post infection with CA/09. (**B**). The inflammation score of day 6 post infection with CA/09. (**D**–**K**) Representative images of inflammatory infiltration at day 3 post infection in mice vaccinated with (**D**) Bris/18, (**E**) Y2, (**F**) Y4, (**G**) CA/09, (**H**) P1, (**I**) X6, (**J**) Bris/07, and (**K**) PBS. (**M**–**T**) Representative images of inflammatory infiltration at day 6 post infection in mice vaccinated with (**M**) Bris/18, (**N**) Y2, (**O**) Y4, (**P**) CA/09, (**Q**) P1, (**R**) X6, (**S**) Bris/07, and (**T**) PBS. (**C**,**L**) Normal mouse lung (no vaccination or infection). Each slide was screened under 20X magnification (scale bar indicated 200 µm) and Inflammation scores are determined as follows: 0 = unremarkable; 1 = <25% of the tissue affected; 2 = 25–50% of the tissue affected; 3 = 50–75% of the tissue affected; 4 = >75 of the tissue affected. One-way ANOVA was used to analyze statistical differences of lung inflammation scores by GraphPad Prism 9 software (GraphPad, San Diego, CA, USA). A *p* value less than 0.05 was defined as statistically significant (*, *p* < 0.05; **, *p* < 0.01; ***, *p* < 0.001; ****, *p* < 0.0001).

Inflammation was correlated with lung injury level. Inflammatory cell infiltration was found in all mice after infection, but significantly more inflammatory cells infiltrated the lungs of mice vaccinated with the Bris/07 VLP vaccine than mice vaccinated with other vaccines or PBS, on day 3 or day 6 post infection (Figure 7A,B). However, there is a trend that mice vaccinated with Bris/18, Y2, CA/09 VLPs, or PBS had slightly increased inflammatory cell infiltration in their lungs on day 6 post infection compared to those on day 3 post infection (Figure 7D–G,K); while the inflammation in mice vaccinated with other vaccines either maintained that level (Figure 7I,J,R,S) or started to decline on day 6 post infection (Figure 7H,Q).

## 5. Discussion

There have been four known influenza virus pandemics that have occurred since 1918, and two of them were caused by subtype H1N1 influenza A viruses. Since the introduction of the H1N1pdm09 virus, the causative agent of the latest influenza virus pandemic, H1N1pdm09-like viruses have circulated seasonally causing illnesses, hospitalizations, and deaths [35,36]. Most of the currently circulating H1N1 influenza viruses are similar to the H1N1pdm09 virus; however, the continual antigenic changes allow the viruses to evade herd immunity through a number of mechanisms, such as neutralizing antibody evasion, viral fitness alternation, and receptor preference variation [37,38]. Moreover, the cross-species transmission from avian and swine viruses to the human population can lead to antigenic shift, enhancing the risk of future pandemics [39].

Therefore, it is necessary to explore improved H1N1 influenza virus vaccines that could induce broadly protective immune responses against multiple stains of co-circulating H1N1 influenza viruses within a season, as well as against newly emerging viral strains. Previously, our group designed two H1N1 COBRA vaccine candidates, P1 and X6, which elicited broadly reactive antibodies with HAI activities against pandemic and/or seasonal H1N1 viruses spanning from 1934 to 2013 [12], that provided efficient protection from CA/09 virus (H1N1pdm09) challenge in both mouse and ferret models [12]. However, these two COBRA HA vaccines were generated based on the HA sequences of H1N1 viruses isolated before 2012, and did not include any sequences that have emerged since then. Therefore, in order to keep up with the natural antigenic drift of H1N1 viruses, the COBRA HA vaccines were brought up to date.

In this study, two next generation H1N1 COBRA HA vaccines, Y2 and Y4, were designed using a seasonal-based COBRA methodology [7] and were derived from HA sequences of H1N1 influenza viruses isolated from 2013 to 2019. These vaccine antigens provided 100% protection against both CA/09 and Bris/18 H1N1 virus infection by preventing morbidity and mortality in two different strains of mice. These two vaccines also elicited protective immunity as efficiently as the homologously matched wild-type HA vaccines against CA/09 and Bris/18 virus infection. In contrast, the historic COBRA HA vaccines, P1 and X6, protected mice from CA/09 virus infection, but did not effectively protect animals against a Bris/18 virus challenge. Although 60% of mice vaccinated with P1 HA survived Bris/18 virus challenge, their body weights dramatically dropped, nearing the humane endpoint by day 6 post infection.

HAI is the primary assay for titrating quantitative antibody titers against influenza viruses [40]. The varying HAI antibody titers elicited by different vaccines partially explained their varied effectiveness during virus infection. The HAI positive antibodies elicited by wild-type CA/09 and Bris/18 HA vaccines elicited highly cross-reactive antibodies with each other, which could provide protection from infection with each of these two viruses. Mice vaccinated with COBRA Y2 and Y4 HA proteins elicited the highest HAI titers against viruses isolated after 2009 and the HAI positive antibody titers against recent circulating strains (Bris/18 and Guangdong/19) that are significantly higher than that detected in the other vaccinated mice. There was no expectation that this same antisera would have HAI titers against any seasonal-like viruses. In contrast, antisera collected from P1-vaccinated mice had high HAI titer against CA/09 virus, but its titer was lower against the Bris/18 and Guangdong/19 viruses. Consistent with the HAI titers, neutralizing antibody assays further confirmed that the antibodies elicited by Y2 and Y4 had high neutralizing activities against both CA/09 and Bris/18 viruses, while the antibodies elicited by P1 did not have neutralizing activity against Bris/18. Interestingly, X6-vaccinated mice did not have any HAI titers, nor neutralizing antibody titers against both CA/09 and Bris/18 viruses, but still had 80% survival rate after CA/09 virus infection. This protection may due to the significantly high amount of stalk-binding antibodies induced in X6-vaccinated mice.

The dominant immune responses against the influenza virus HA protein are directed towards the head of the HA, specifically to defined antigenic sites that surround the receptor binding pocket [41]. It is likely that these head-specific antibodies neutralize viruses by inhibiting receptor binding [42]. However, the immune response could also be directed to the stalk of the HA protein. Stalk-binding antibodies neutralize viruses through distinct post-binding mechanisms [42]. Stalk-specific neutralizing antibodies are typically less abundant and less potent than antibodies specific for the globular head [43,44,45,46]. However, the stem-binding antibodies that do not have neutralizing activities are potent inducers of antibody-dependent cell-mediated cytotoxicity (ADCC), which is essential for optimal protection in vivo [47,48], while head-specific HAI+ antibodies do not elicit ADCC [47]. This could partially explain why 80% of the mice vaccinated with X6 survived from CA/09 infection, and 60% of mice vaccinated with P1 survived from Bris/18 virus infection, even though no neutralizing antibodies or HAI positive antibodies were detected in their antisera.

The robust inflammatory responses in the lungs following infection are critical for efficient virus clearance, but it is not always beneficial to the infected host [49]. In the context of influenza virus infection, lung inflammatory responses are a double-edged sword: on one hand, immune effector recruitment is necessary for eliminating virus-infected cells, and on the other hand, the excessive inflammatory cell accumulation and the subsequent “cytokine storm” also hinder lung function, causing more severe influenza infection, lung disease, and in some cases, death of the host [50,51]. The fine-turning of the inflammatory response is pivotal not only efficient virus clearance, but also for reducing the virus infection associated lung tissue damage [51].

Alveoli are the terminal end of the respiratory system, where gas exchange occurs between alveolar air and the blood of the pulmonary capillaries [52,53]. The alveolar septum has thin walls for gas exchange, but they become thicker during influenza virus infection, which reduces the gas-exchange along with elevating the fluid exchange between the capillary and interstitium [52,54,55,56]. In this study, COBRA Y2- and Y4 HA VLP-vaccinated mice had moderate inflammation in their lungs on day 3 post infection, and the inflammation did not increase at day 6 post infection. As a result, lung injuries were significantly lower in these mice, including reduced proteinaceous debris filling the airspace, and reduced alveolar septal thickening. However, no statistically significant differences were observed in regards to neutrophil infiltration in the alveolae between the VLP-vaccinated and the PBS-vaccinated groups. A similar phenomenon was also observed in the CA/09-, Bris/18-, and P1 VLP-vaccinated mice after CA/09 infection. However, the mice vaccinated with X6 VLPs had a higher level of inflammation from day 3 through day 6 post infection, resulting in more lung injury compared to other mice, except for the Bris/07 VLP-vaccinated mice, which also had high viral titers in the lung and increased body weight loss. The Bris/07 VLP- and mock-vaccinated mice had the most inflammation in their lungs during the infection period, and this overwhelming inflammation could not be controlled, resulting in severe lung injury that resulted in animals succumbing to the disease.

In summary, the two next-generation H1N1 COBRA HA antigens, Y2 and Y4, were designed using a next generation seasonal-based COBRA methodology. These two vaccines protected animals from infection of both pandemic CA/09 virus and the recent circulating Bris/18 virus by preventing morbidity and mortality. Moreover, the antibodies elicited by these two COBRA HA antigens displayed broader HAI activity against all the pandemic-like viruses isolated since 2009, and the HAI titers were elevated against the most recent representative circulating viruses, including Bris/18 and Guangdong/19. In addition, Y2 and Y4 VLPs induced antibodies against the CA/09 and Bris/18 viruses that are likely head-specific neutralizing antibodies, which inhibit viral replication in lung tissues to low or undetectable levels, therefore resulting in a well-controlled inflammatory response and decreased lung injury.

## Figures and Tables

**Table 1:** Multiple alignment of all full-length HA sequences included in this study.

	1	10	20	30	40	50	60	
	|	|	|	|	|	|	|	
CA/09 MKAILVVLLYTFATANADTLCIGYHANNSTDTVDTVLEKNVTVTHSVNLLEDKHNGKLCK Bris/18 MKAILVVLLYTFTTANADTLCIGYHANNSTDTVDTVLEKNVTVTHSVNLLEDKHNGKLCK Y2 MKAILVVLLYTFTTANADTLCIGYHANNSTDTVDTVLEKNVTVTHSVNLLEDKHNGKLCK Y4 MKAILVVLLYTFTTANADTLCIGYHANNSTDTVDTVLEKNVTVTHSVNLLEDKHNGKLCK P1 MKARLLVLLCALAATDADTICIGYHANNSTDTVDTVLEKNVTVTHSVNLLEDSHNGKLCK X6 MEARLLVLLCAFAATNADTICIGYHANNSTDTVDTVLEKNVTVTHSVNLLEDSHNGKLCL Bris/07 MKVKLLVLLCTFTATYADTICIGYHANNSTDTVDTVLEKNVTVTHSVNLLENSHNGKLCL								
	61	70	80	90	100	110	120	
	|	|	|	|	|	|	|	
CA/09 LRGVAPLHLGKCNIAGWILGNPECESLSTASSWSYIVETPSSDNGTCYPGDFIDYEELRE Bris/18 LGGVAPLHLGKCNIAGWILGNPECESLSTARSWSYIVETSNSDNGTCYPGDFINYEELRE Y2 LRGVAPLHLGKCNIAGWILGNPECESLSTASSWSYIVETSNSDNGTCYPGDFINYEELRE Y4 LRGVAPLHLGKCNIAGWILGNPECESLSTARSWSYIVETSNSDNGTCYPGDFINYEELRE P1 LKGIAPLQLGKCNIAGWLLGNPECESLLSARSWSYIVETPNSENGTCYPGDFIDYEELRE X6 LKGIAPLQLGNCSVAGWILGNPECELLISKESWSYIVETPNPENGTCYPGYFADYEELRE Bris/07 LKGIAPLQLGNCSVAGWILGNPECELLISKESWSYIVEKPNPENGTCYPGHFADYEELRE								
	121	130	140	150	160	170	180	
	|	|	|	|	|	|	|	
CA/09 QLSSVSSFERFEIFPKTSSWPNHDSNKGVTAACPHAGAKSFYKNLIWLVKKGNSYPKLSK Bris/18 QLSSVSSFERFEIFPKTSSWPNHDSNKGVTAACPHAGAKSFYKNLIWLVKKGNSYPKLNQ Y2 QLSSVSSFERFEIFPKTSSWPNHDSNKGVTAACPHAGAKSFYKNLIWLVKKGNSYPKLSQ Y4 QLSSVSSFERFEIFPKTSSWPNHDSNKGVTAACPHAGAKSFYKNLIWLVKKGNSYPKLNQ P1 QLSSVSSFERFEIFPKESSWPNHNTTKGVTAACSHAGKSSFYRNLLWLTKKGGSYPKLSK X6 QLSSVSSFERFEIFPKESSWPNH-TVTGVSASCSHNGKSSFYRNLLWLTGKNGLYPNLSK Bris/07 QLSSVSSFERFEIFPKESSWPNH-TVTGVSASCSHNGESSFYRNLLWLTGKNGLYPNLSK								
	181	190	200	210	220	230	240	
	|	|	|	|	|	|	|	
CA/09 SYINDKGKEVLVLWGIHHPSTSADQQSLYQNADAYVFVGSSRYSKKFKPEIAIRPKVRDQ Bris/18 TYINDKGKEVLVLWGIHHPPTTADQQXLYQNADAYVFVGTSRYSKKFKPEIATRPKVRDQ Y2 SYINDKGKEVLVLWGIHHPSTTADQQSLYQNADAYVFVGTSRYSKKFKPEIAIRPKVRDQ Y4 TYINDKGKEVLVLWGIHHPSTTADQQSLYQNADAYVFVGTSRYSKKFKPEIATRPKVRDQ P1 SYVNNKGKEVLVLWGVHHPSTSTDQQSLYQNENAYVSVVSSNYNRRFTPEIAERPKVRGQ X6 SYANNKEKEVLVLWGVHHPPNIGDQRALYHTENAYVSVVSSHYSRKFTPEIAKRPKVRDQ Bris/07 SYANNKEKEVLVLWGVHHPPNIGNQKALYHTENAYVSVVSSHYSRKFTPEIAKRPKVRDQ								
	241	250	260	270	280	290	300	
	|	|	|	|	|	|	|	
CA/09 EGRMNYYWTLVEPGDKITFEATGNLVVPRYAFAMERNAGSGIIISDTPVHDCNTTCQTPK Bris/18 EGRMNYYWTLVEPGDKITFEATGNLVVPRYAFTMERNAGSGIIISDTPVHDCNTTCQTAE Y2 EGRMNYYWTLVEPGDKITFEATGNLVVPRYAFTMERNAGSGIIISDTPVHDCNTTCQTPE Y4 EGRMNYYWTLVEPGDKITFEATGNLVVPRYAFTMERNAGSGIIISDTPVHDCNTTCQTPE P1 AGRMNYYWTLLEPGDTIIFEATGNLIAPWYAFALSRGSGSGIITSNASMHECNTKCQTPQ X6 EGRINYYWTLLEPGDTIIFEANGNLIAPRYAFALSRGFGSGIITSNAPMDECDAKCQTPQ Bris/07 EGRINYYWTLLEPGDTIIFEANGNLIAPRYAFALSRGFGSGIINSNAPMDKCDAKCQTPQ								
	301	310	320	330	340	350	360	
	|	|	|	|	|	|	|	
CA/09 GAINTSLPFQNIHPITIGKCPKYVKSTKLRLATGLRNIPSIQSRGLFGAIAGFIEGGWTG Bris/18 GAINTSLPFQNVHPVTIGKCPKYVKSTKLRLATGLRNVPSIQSRGLFGAIAGFIEGGWTG Y2 GAINTSLPFQNVHPITIGKCPKYVKSTKLRLATGLRNVPSIQSRGLFGAIAGFIEGGWTG Y4 GAINTSLPFQNVHPITIGKCPKYVKSTKLRLATGLRNVPSIQSRGLFGAIAGFIEGGWTG P1 GAINSSLPFQNIHPVTIGECPKYVRSTKLRMVTGLRNIPSIQSRGLFGAIAGFIEGGWTG X6 GAINSSLPFQNVHPVTIGECPKYVRSAKLRMVTGLRNIPSIQSRGLFGAIAGFIEGGWTG Bris/07 GAINSSLPFQNVHPVTIGECPKYVRSAKLRMVTGLRNIPSIQSRGLFGAIAGFIEGGWTG								
	361	370	380	390	400	410	420	
	|	|	|	|	|	|	|	
CA/09 MVDGWYGYHHQNEQGSGYAADLKSTQNAIDEITNKVNSVIEKMNTQFTAVGKEFNHLEKR Bris/18 MVDGWYGYHHQNEQGSGYAADLKSTQNAIDKITNKVNSVIEKMNTQFTAVGKEFNHLEKR Y2 MVDGWYGYHHQNEQGSGYAADLKSTQNAIDKITNKVNSVIEKMNTQFTAVGKEFNHLEKR Y4 MVDGWYGYHHQNEQGSGYAADLKSTQNAIDKITNKVNSVIEKMNTQFTAVGKEFNHLEKR P1 MIDGWYGYHHQNEQGSGYAADQKSTQNAINGITNKVNSVIEKMNTQFTAVGKEFNNLEKR X6 MVDGWYGYHHQNEQGSGYAADQKSTQNAINGITNKVNSVIEKMNTQFTAVGKEFNKLERR Bris/07 MVDGWYGYHHQNEQGSGYAADQKSTQNAINGITNKVNSVIEKMNTQFTAVGKEFNKLERR								
	421	430	440	450	460	470	480	
	|	|	|	|	|	|	|	
CA/09 IENLNKKVDDGFLDIWTYNAELLVLLENERTLDYHDSNVKNLYEKVRSQLKNNAKEIGNG Bris/18 IENLNKKVDDGFLDIWTYNAELLVLLENERTLDYHDSNVKNLYEKVRNQLKNNAKEIGNG Y2 IENLNKKVDDGFLDIWTYNAELLVLLENERTLDYHDSNVKNLYEKVRNQLKNNAKEIGNG Y4 IENLNKKVDDGFLDIWTYNAELLVLLENERTLDYHDSNVKNLYEKVRNQLKNNAKEIGNG P1 MENLNKKVDDGFLDIWTYNAELLVLLENERTLDFHDSNVKNLYEKVKSQLRNNAKEIGNG X6 MENLNKKVDDGFLDIWTYNAELLVLLENERTLDFHDSNVKNLYEKVKSQLKNNAKEIGNG Bris/07 MENLNKKVDDGFIDIWTYNAELLVLLENERTLDFHDSNVKNLYEKVKSQLKNNAKEIGNG								
	481	490	500	510	520	530	540	
	|	|	|	|	|	|	|	
CA/09 CFEFYHKCDNTCMESVKNGTYDYPKYSEEAKLNREEIDGVKLESTRIYQILAIYSTVASS Bris/18 CFEFYHKCDNTCMESVKNGTYDYPKYSEEAKLNREKIDGVKLESTRIYQILAIYSTVASS Y2 CFEFYHKCDNTCMESVKNGTYDYPKYSEEAKLNREKIDGVKLESTRIYQILAIYSTVASS Y4 CFEFYHKCDNTCMESVKNGTYDYPKYSEEAKLNREKIDGVKLESTRIYQILAIYSTVASS P1 CFEFYHKCDNECMESVKNGTYDYPKYSEESKLNREKIDGVKLESMGVYQILAIYSTVASS X6 CFEFYHKCNNECMESVKNGTYDYPKYSEESKLNREKIDGVKLESMGVYQILAIYSTVASS Bris/07 CFEFYHKCNDECMESVKNGTYDYPKYSEESKLNREKIDGVKLESMGVYQILAIYSTVASS								
			541	550	560			
			|	|	|			
CA/09 LVLVVSLGAISFWMCSNGSLQCRICI Bris/18 LVLVVSLGAISFWMCSNGSLQCRICI Y2 LVLVVSLGAISFWMCSNGSLQCRICI Y4 LVLVVSLGAISFWMCSNGSLQCRICI P1 LVLLVSLGAISFWMCSNGSLQCRICI X6 LVLLVSLGAISFWMCSNGSLQCRICI Bris/07 LVLLVSLGAISFWMCSNGSLQCRICI								

## Data Availability

The data presented in this study are available on request from the corresponding author.
