# Correction: Huang et al. Next Generation of Computationally Optimized Broadly Reactive HA Vaccines Elicited Cross-Reactive Immune Responses and Provided Protection against H1N1 Virus Infection. *Vaccines* 2021, *9*, 793

**DOI:** 10.3390/vaccines12111283

**Published:** 2024-11-15

**Authors:** Ying Huang, Monique S. França, James D. Allen, Hua Shi, Ted M. Ross

**Affiliations:** 1 Center for Vaccines and Immunology, University of Georgia, Athens, GA 30602, USA; yhuang0@uga.edu (Y.H.); jdallen@uga.edu (J.D.A.); hua.shi1@uga.edu (H.S.); 2 Poultry Diagnostic and Research Center, Department of Population Health, University of Georgia, Athens, GA 30602, USA; mfranca@uga.edu; 3 Department of Infectious Diseases, University of Georgia, Athens, GA 30602, USA

The authors would like to make the following corrections to this published paper [1].

In the original publication, there was a typographical error in Figure 1E. The CA-09 survival rate should be 100%, instead of 1000%. The corrected Figure 1 is attached below.

In the original publication, due to oversight, there was a duplication of the panel in Figure 6, i.e., Figure 6D Bris/18 and 6G CA/09. The corrected Figure 6 is attached below.

Some typographical and grammar errors in Figures 1 and 6 legends are also corrected.

The authors state that the scientific conclusions are unaffected. These corrections were approved by the Academic Editor. The original publication has also been updated.

## Figures and Tables

**Figure 1:**
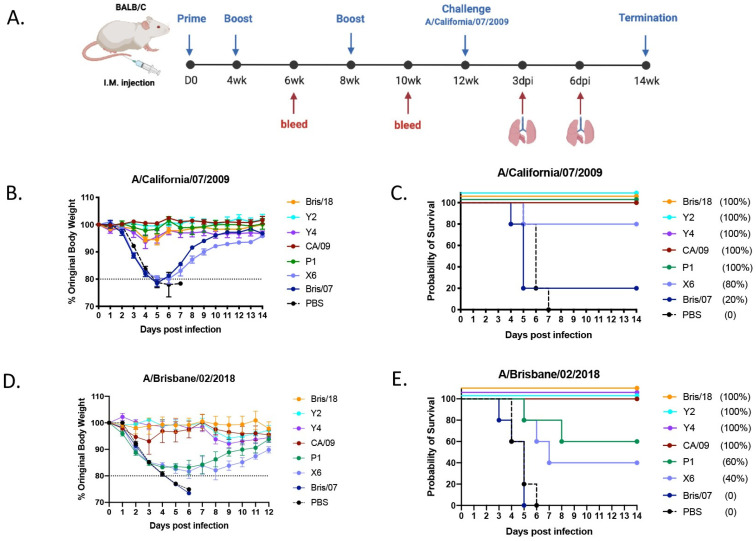
Schematic of animal study. (**A**) Animal study outline: Eighty-eight BALB/c mice (*n* = 11) were intramuscularly vaccinated with COBRA or wild-type HA VLP vaccines formulated with AddaVax adjuvant at weeks 0, 4, and 8. At weeks 6 and 10 post vaccination, blood was collected, and the sera were separated for analysis. At week 12, all mice were inoculated with 5 × 10^4^ PFU of A/California/07/2009 H1N1 virus intranasally, lung tissues (*n* = 3/group) were harvested on days 3 and 6 post infection and evaluated for histopathology and virus titration. (**B**) Body weight loss of mice post infection: The mice were observed for clinical signs for 14 days, and their body weight was recorded daily post infection. The dotted line indicates 80% of their body weights on D0 post infection. (**C**) Survival cure after infection with A/California/07/2009 virus. Another 64 DBA/2J mice were intramuscularly vaccinated with COBRA or wild-type rHA vaccines formulated with AddaVax adjuvant using the same vaccination regimen mentioned above. At week 12, all mice were intranasally infected with 8.75 × 10^6^ PFU of A/Brisbane/02/2018 H1N1 virus. (**D**) Body weight loss curve of DBA/2J mice after infection with A/Brisbane/02/2018 H1N1 virus. (**E**) Survival cure after infection with A/Brisbane/02/2018 virus.

**Figure 6:**
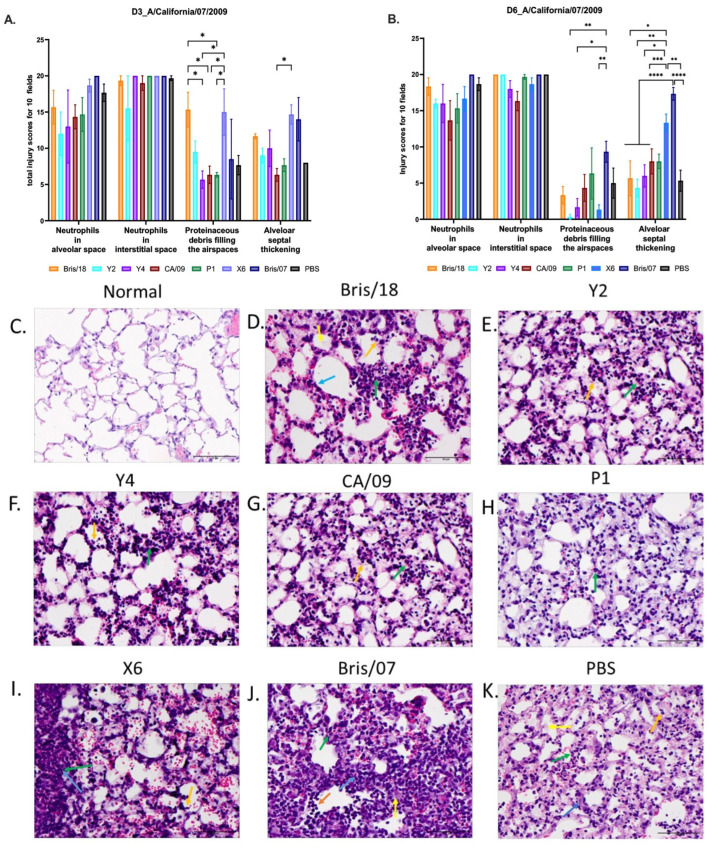
Lung injury in mice after A/California/09/2007 virus infection. Three mice from each group were euthanized on day 3 (**A**) and day 6 post infection (**B**). The left lungs were inflated with 10% buffered formalin and then embedded into paraffin blocks. H&E staining was performed on 3 sectional slides for each mouse lung sample. Neutrophils in alveolar space, neutrophils in interstitial space, proteinaceous debris filling the airspaces, and alveolar septal thickening were assessed. Each parameter was evaluated in 10 random fields under 40× magnification (scale bar indicated 50 µm), and then, the total injury scores of 10 fields were determined. Scoring system: Neutrophils in alveolar space (indicated with orange arrows): 0 = none, 1 = 1–5, 2 = >5; neutrophils in interstitial space (indicated with green arrows): 0 = none, 1 = 1–5, 2 = >5; proteinaceous debris filling the airspaces (indicated with yellow arrows): 0 = none, 1 = 1, 2 = >1; alveolar septal thickening (indicated with blue arrows): 0 = <2×, 1 = 2×–4×, 2 = >4×. (**C**) Normal mouse left lung (no vaccination or infection). (**D**–**K**) Representative images of alveolar septal thickening on day 6 post infection in mice vaccinated with (**D**) Bris/18, (**E**) Y2, (**F**) Y4, (**G**) CA/09, (**H**) P1, (**I**) X6, (**J**) Bris/07, and (**K**) PBS. The data are presented as absolute mean plus SEM. A one-way ANOVA was used to analyze the statistical differences of the lung injury scores using GraphPad Prism 9 software (GraphPad, San Diego, CA, USA). A *p* value less than 0.05 was defined as statistically significant (*, *p* < 0.05; **, *p* < 0.01; ***, *p* < 0.001; ****, *p* < 0.0001).
